# A study of the prognosis of patients with limited-stage small cell lung cancer who did or did not receive prophylactic cranial irradiation after effective chemoradiotherapy

**DOI:** 10.3389/fonc.2023.1118371

**Published:** 2023-03-23

**Authors:** Qing Wu, Mengyuan Chen, Fang Peng, Qun Zhang, Yue Kong, Yong Bao, Yujin Xu, Xiao Hu, Ming Chen

**Affiliations:** ^1^ The Second Clinical Medical College of Zhejiang Chinese Medical University, Hangzhou, Zhejiang, China; ^2^ Department of Thoracic Radiotherapy, Zhejiang Cancer Hospital, Institute of Basic Medicine and Cancer (IBMC), Chinese Academy of Sciences, Zhejiang Key Laboratory of Radiation Oncology, Hangzhou, Zhejiang, China; ^3^ Department of Radiotherapy, The First Affiliated Hospital of Sun Yat-sen University, Guangzhou, Guangdong, China; ^4^ Department of Radiotherapy, Sun Yat-sen University Cancer Center, Guangzhou, Guangdong, China

**Keywords:** small cell lung cancer, limited-stage, prophylactic cranial irradiation, brain metastases, study on the prognosis

## Abstract

**Objective:**

To investigate the prognosis of patients with LS-SCLC who responded to chest chemoradiotherapy but did not receive PCI.

**Methods:**

A retrospective analysis was conducted on LS-SCLC patients who had achieved complete remission (CR) or partial remission (PR) after definitive chemoradiotherapy but did not receive PCI. The survival rates were calculated using Kaplan–Meier method. The prognosis was analyzed using Cox proportional hazard regression model. The main endpoint was OS.

**Results:**

Of the 500 patients with LS-SCLC admitted between June 2002 and January 2018, 327 achieved CR or PR after definitive chest chemoradiotherapy, 103 did not receive PCI, and 63 of them developed brain metastases (BM). The 1-year and 3-year OS rates in PCI group were 87.5% and 42.3% respectively, versus 70.4% and 20.9% for non-PCI group(P=0.002). The median survival time after BM was 8.7 months (range: 0.3-48.7), and 3-year OS rate was 15.0%, the median survival time of patients without BM was 20.1 months (range: 2.9-79.4), and 3-year OS was 33.4% (P=0.014). Patients with BM were subsequently treated with palliative therapy. Multivariate analysis showed that compared with no treatment, brain radiotherapy alone (HR: 0.131, 95%CI: 0.035-0.491, P=0.003) and radiotherapy combined with chemotherapy (HR: 0.039, 95%CI: 0.008-0.194, P<0.001) significantly reduced the risk of death. Multiple BM (HR: 2.391, 95%CI: 1.082-5.285, P=0.031) was an independent adverse prognostic factor for OS.

**Conclusion:**

LS-SCLC patients who achieved good response after chest chemoradiotherapy without receiving PCI were prone to develop BM and have a poor prognosis. Multiple BM was an independent adverse prognostic factor. PCI remains the standard of care for LS-SCLC patients.

## Introduction

Small cell lung cancer (SCLC) accounts for about 15% of all lung cancers, and limited-stage (LS) patients account for about 30% of all SCLC (1, 2). The current standard treatment is chest radiotherapy combined with concurrent chemotherapy for most LS-SCLC patients (3). SCLC prone to develop brain metastasis (BM), and patients with BM have a poor prognosis. A meta-analysis showed that prophylactic cranial irradiation (PCI) significantly reduced the incidence of BM and increased 3 years OS for LS-SCLC patients who had achieved complete remission (CR) after thoracic chemoradiotherapy, compared with the control group (4). Based on this study, at present, PCI is recommended for LS-SCLC patients who achieve good response (5). However, PCI might potentially detriment neurocognitive function, especially in elder patients (6).

In clinical practice, some patients achieved CR or partial remission (PR) of tumor after chest chemoradiotherapy but refused to receive PCI with the fear of neurotoxicity and unfortunately developed BM. This study aimed to investigate the prognosis of these patients who did not receive PCI.

## Patients and methods

### Patient eligibility

Between June 2002 and January 2018, patients who were diagnosed as LS-SCLC, had achieved CR or PR after definitive chemoradiotherapy but did not receive PCI were included in this study. The following patients were excluded: diagnosed as mixed SCLC, received surgical treatment except pathological biopsy, have a history of other malignant tumors, patients who were lost to follow-up after treatment. Demographic variables, which were obtained from medical records on the electronic database of Zhejiang Cancer Hospital, included age, gender, Karnofsky Performance Status (KPS), and so on. All data access was approved by the Ethics Committee of Zhejiang Cancer Hospital (IRB-2022-758).

### Treatment modalities

Chemotherapy consisted of etoposide 100mg/m^2^ d1-3, cisplatin 80mg/m^2^ d1 or 25mg/m^2^ d1-3, or carboplatin AUC=5 d1, repeated every 3 weeks for 4-6 cycles.

All patients received accelerated hyperfractionated radiotherapy consisted of 1.5 Gy twice daily in 30 fractions to 45 Gy, and the interval between fractions was ≥6 hours.

Patients received chest and abdominal enhanced CT and brain enhanced MRI 4-6 weeks after chest chemoradiotherapy, for those who had achieved CR or PR, PCI was recommended. The prescribed dose of PCI was 25Gy in 10 fractions.

### Follow-up scheme

Patients were reviewed every 3 months in 2 years after chemoradiotherapy, every 6 months in 3 to 5 years, and then once every year after 5 years. Physical examination, enhanced CT of chest and upper abdomen were performed routinely. The frequency of brain MRI (first choice) or brain CT or positron emission computed tomography was at the discretion of the physician. Brain metastases were determined by brain MRI or CT imaging features. After treatment of BM, patients were reviewed every 2 months in the first year, then every 3 to 4 months in the second to third year, and every 6 months thereafter.

### Survival and statistical analysis

In this study, OS was defined as the time from the end of chemoradiotherapy to death or last follow-up. Statistics and graphing were conducted using SPSS 25.0 and GraphPad Prism 8.0 software. The two-tailed t-test was used for continuous variables. Mann-Whitney U test was used to compare non-normally distributed data. Categorical variables were compared using the X^2^ test. The survival outcomes were calculated using the Kaplan–Meier method. The Cox proportional models were applied for univariate and multivariate prognostic analyses. Propensity score-matching (PSM) was performed using the R MatchIt package for Windows version. A two-tailed value of p < 0.05 denoted statistical significance.

### PSM

The propensity score was calculated using multivariable logistic regression to model a dichotomous outcome of PCI or non-PCI. In an initial analysis, patients in the PCI and non-PCI groups were compared based on age, gender, KPS, AJCC Staging and curative effect. The subjects were 1:1 matched by the estimated propensity score with a caliper of 0.02 of the logit of the propensity score.

## Results

### Patient characteristics

From June 2002 to January 2018, 500 patients with LS-SCLC were admitted, of which 362 (72.4%) received definitive chest radiotherapy and chemotherapy. According to Response Evaluation Criteria In Solid Tumors (RECIST), 137 cases (37.8%) had achieved CR and 190 cases (52.5%) had achieved PR after chemoradiotherapy. Among the patients who achieved CR and PR, 103 cases (31.5%) did not receive PCI. 63 patients (61.2%) developed BM, of which, 19 patients (30.2%) had BM detected by brain MRI before PCI. By the last follow-up date (June 25, 2022), a total of 172 patients had follow-up data, of whom 98 had received PCI and 74 had not received PCI. The matching process resulted in a final cohort of 96 patients. Each cohort comprised 48 patients. The eligible patients in two groups were similar in terms of age (median: 64 vs. 62 years, respectively), gender, KPS (median: 90 vs. 90, respectively), AJCC stage, and curative effect (**Table 1**).

**Table 1:** Characteristics of propensity score-matched patients.

Variables	PCI, n (%)	Non-PCI, n (%)	*P*
Age(years)			0.195
Median (range)	64 (43-81)	62 (48-82)	
Gender			1.000
Male	41 (85.4)	41 (85.4)	
Female	7 (14.6)	7 (14.6)	
KPS			
Median (range)	90 (80-100)	90 (80-100)	
Loss weight			0.312
<5%	37 (77.1)	37 (77.1)	
5-10%	10 (20.8)	7 (14.6)	
10-20%	1 (2.1)	4 (8.3)	
AJCC			1.000
IIB	3 (6.3)	3 (6.3)	
IIIA	21 (43.8)	21 (43.8)	
IIIB	24 (50.0)	24 (50.0)	
Curative effect			0.399
CR	2 (4.2)	4 (8.3)	
PR	46(95.8)	44 (91.7)	

KPS, Karnofsky performance status; AJCC, American Joint Committee on Cancer; PCI, prophylactic cranial irradiation; CR, complete remission; PR, partial remission.

In the 74 patients who did not receive PCI, 40 patients developed brain metastases. These BM patients had regular follow-up data after treatment were analyzed, as shown in **Table 2**. The median interval time between the discovery of BM and the initial treatment was 5.6 months (range: 2.9-26.6 months), and the median interval time between the discovery of BM and the completion of chemoradiotherapy was 1.6 months (range: 0.2-24.2 months). Among the 9 patients who received radiotherapy combined with chemotherapy, 5 patients received whole brain radiotherapy (WBRT), 3 patients received WBRT plus focal radiation boost, and 1 patient received X-knife. Among the 23 patients who received brain radiotherapy alone, 7 patients received WBRT, 9 patients received WBRT plus focal radiation boost, 1 patient received γ-knife therapy, and radiotherapy technique in 6 patients was unknown. Four patients received chemotherapy alone. Four patients did not receive any treatment.

**Table 2:** Clinical characteristics of 40 patients with BM.

Variables	Classification	Number	Proportion (%)
Age (years)	65-75	6	15.0
	55-64	25	62.5
	≤54	9	22.5
Gender	Male	35	87.5
	Female	5	12.5
KPS	≥90	22	55.0
	<90	18	45.0
Loss weight	<5%	29	72.5
	5-10%	8	20.0
	10-20%	3	7.5
AJCC	IIB	2	5.0
	IIIA	10	25.0
	IIIB	28	70.0
Brain metastases were found before PCI	Yes	19	47.5
	No	21	52.5
Multiple brain metastases	Yes	27	67.5
	No	13	32.5
Symptomatic brain metastases	Yes	18	45.0
	No	22	55.0
Extracranial disease control	Well controlled	25	62.5
	Out of control	15	37.5
Treatment of brain metastases	Radiotherapy alone	23	57.5
	Chemotherapy alone	4	10.0
	Radiotherapy combined with chemotherapy	9	22.5
	No treatment	4	10.0

KPS, Karnofsky performance status; AJCC, American Joint Committee on Cancer; PCI, prophylactic cranial irradiation.

### Survival

**Figure 1:**
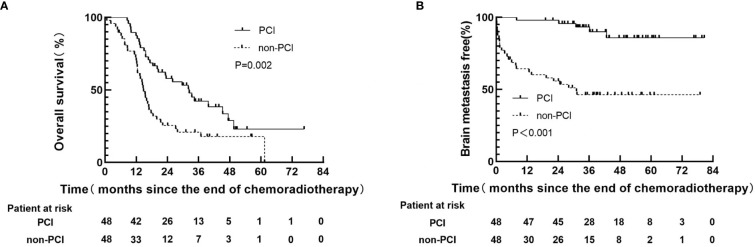
Kaplan-Meier curves following propensity score-matching for overall survival **(A)**, brain metastasis free **(B)**.

By the last follow-up (June 25, 2022), 30(62.5%) patients had died in PCI group, and 39(81.3%) patients had died in non-PCI group, respectively. All of which were related to disease progression. The median follow-up time was 18.4 months (range: 0.7-76.6). The 1-year and 3-year OS rates in PCI group were 87.5% and 42.3% respectively, versus 70.4% and 20.9% for non-PCI group(P=0.002), as shown in **Figure 1A**. The 1-year and 3-year BM-free rates in PCI group were 97.9% and 93.3% respectively, versus 64.4% and 46.4% for non-PCI group(P<0.001), as shown in **Figure 1B**.

**Figure 2:**
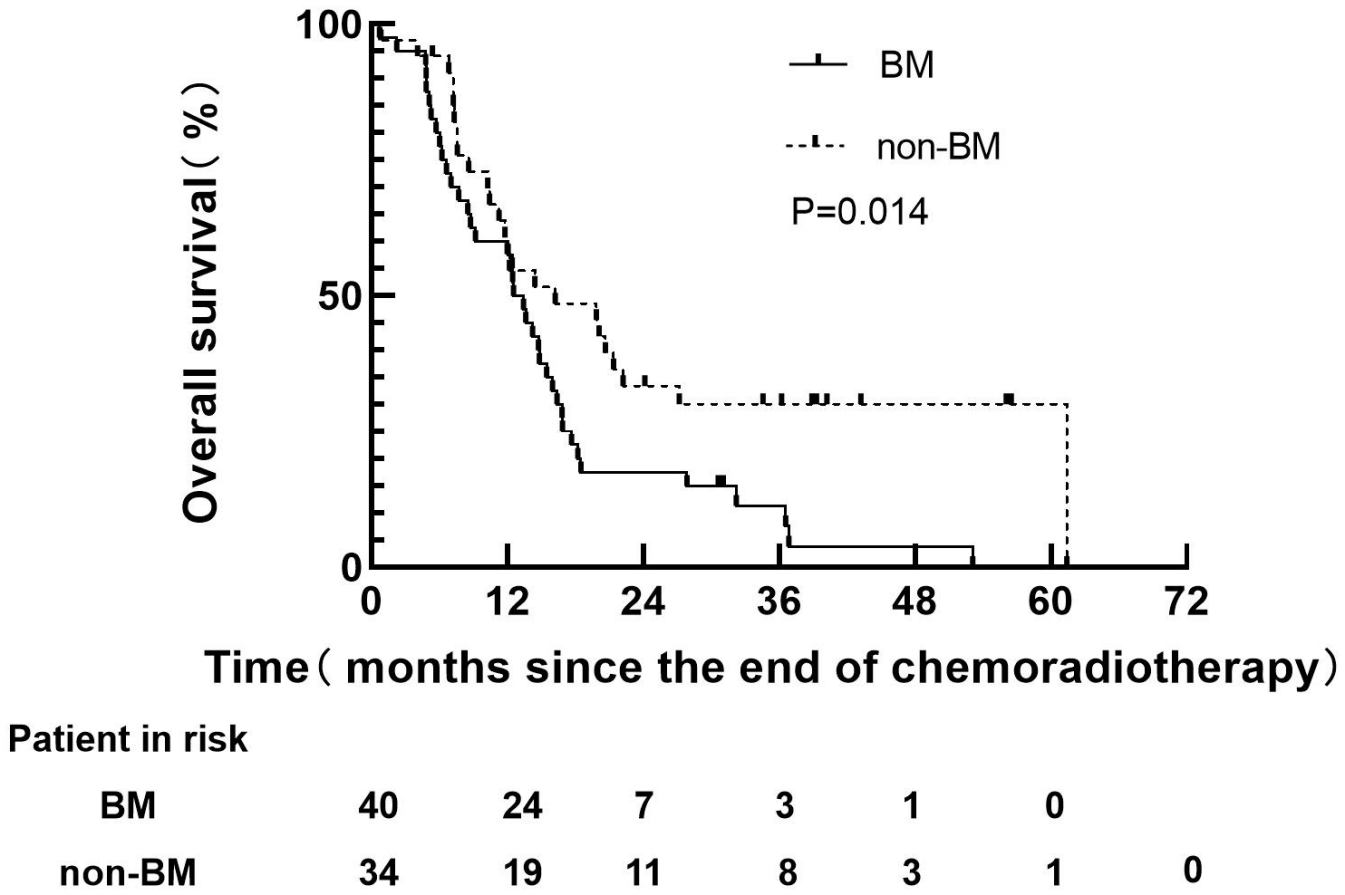
Overall survival of patients in non-PCI group before PSM.

Before PSM, 40 patients with BM in non-PCI group, 38 patients had died, all of which were related to disease progression. However, 34 patients without BM in non-PCI group, 24 patients had died, all of which were related to disease progression. The median follow-up time of 40 patients with BM were 12.5 months (range: 0.7-53.1), and the median survival time was 12.5 months (range: 0.7-53.1). The median follow-up time of 34 patients without BM was 14.4 months (range: 0.8-61.4), and the survival time was 15.3 months (range: 0.8-61.4). The 1-year and 3-years OS rates in BM group were 60.0 and 15.0% respectively, versus 60.7% and 33.4% for non-BM group (P=0.014), as shown in **Figure 2**. The median survival time of BM patients with well controlled and out of controlled extracranial disease were 9.0 months (range: 0.3-48.7) and 6.7 months (range: 0.9-35.2).

**Figure 3:**
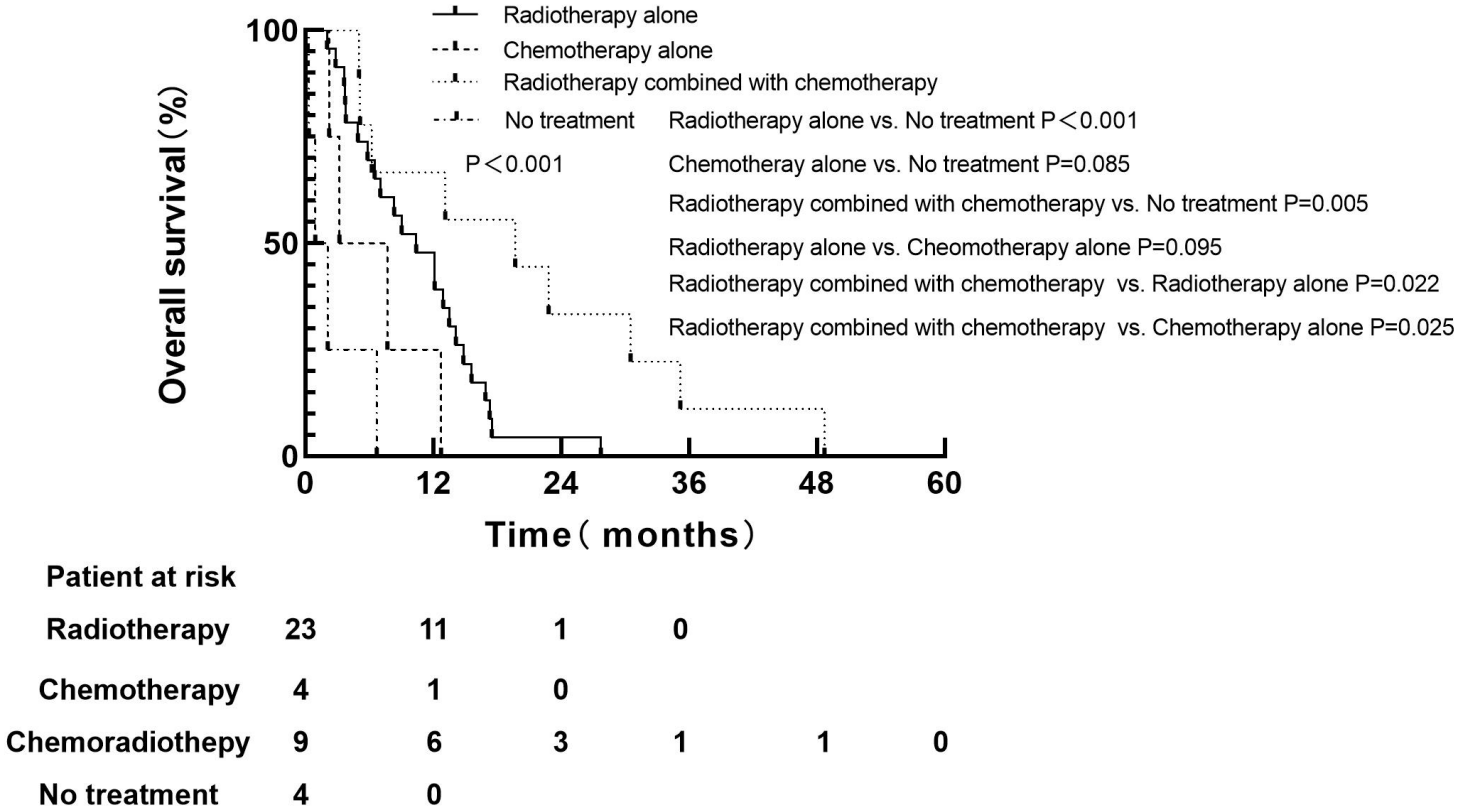
Overall survival based on different treatment groups after BM.

For 74 patients in non-PCI group, univariate and multivariate analysis were used to determine the prognostic value of clinical risk factors affecting BM, as shown in **Table 3**. Patients whose age ≤61 years were more likely to develop BM than patients > 61 years of age(P=0.007). Patients whose KPS <90 were more likely to develop brain metastases than those whose KPS ≥90(P=0.005). For 40 patients with BM in non-PCI group who received palliative treatment, OS was defined as the time from the diagnosis of BM to death or last follow-up. The median survival time of patients who received radiotherapy combined with chemotherapy, radiotherapy alone, chemotherapy alone and no treatment were 19.7 months (range: 5.0-48.7), 10.4 months (range: 2.0-27.7), 7.0 months (range: 2.2-12.7) and 2.0 months (range: 0.3-6.7), respectively. Log-rank test indicated that patients who received radiotherapy alone or chemoradiotherapy both had significantly higher OS rates than untreated patients (chemoradiotherapy vs. untreated, P=0.005; Radiotherapy alone vs. untreated, P < 0.001). There was no significant difference in OS between patients who received chemotherapy alone and no treatment (P=0.085). Compared with monotherapy, combined therapy significantly improved OS (radiotherapy combined with chemotherapy vs. radiotherapy alone, P=0.022; Radiotherapy combined with chemotherapy vs. chemotherapy alone, P=0.025). There was no significant difference in OS between the two monotherapies (radiotherapy alone vs. chemotherapy alone, P=0.095), as shown in **Figure 3**.

**Table 3:** univariate and multivariate analysis of clinical factors of 74 patients in non-PCI group for brain metastases.

Variables	Number	Univariate		Multivariate	
		*HR* (95%*CI*)	*P*	*HR* (95%*CI*)	*P*
Age (years)			0.005		0.007
>61	37	1		1	
≤61	37	2.530(1.315-4.866)		2.468 (1.280-4.757)	
Gender			0.369		–
male	66	1		–	
female	8	1.537(0.601-3.928)		–	
KPS			0.004		0.005
≥90	50	1		1	
<90	24	2.516(1.343-4.710)		2.446 (1.304-4.590)	
Loss weight (%)			0.933		–
<5	55	1		–	
≥5	19	1.030(0.514-2.065)		–	
AJCC					
IIB		1		–	–
IIIA		0.448(0.098-2.045)	0.300	–	
IIIB		1.390(0.331-5.844)	0.653	–	

KPS, Karnofsky performance status; AJCC, American Joint Committee on Cancer; -, uncompared.

Variables such as age, gender, the interval time between BM and the initial treatment, whether multiple BM and the treatment modalities adopted after BM were included in the univariate Cox regression analysis. The results suggested that symptomatic BM, receiving brain radiotherapy alone and receiving radiotherapy combined with chemotherapy were significantly correlated with OS. Variables with P < 0.2 in univariate analysis were included in multivariate Cox regression analysis. The results showed that, compared with no treatment, patients received brain radiotherapy alone (HR: 0.131, 95%CI: 0.035-0.491, P=0.003), radiotherapy combined with chemotherapy (HR: 0.039, 95%CI: 0.008-0.194, P < 0.001) significantly reduced the risk of death. Multiple BM (HR: 2.391, 95%CI: (1.082-5.285), P=0.031) was an independent adverse prognostic factor for OS, as shown in **Table 4**.

**Table 4:** univariate and multivariate analysis of clinical factors for overall survival.

Variables	Number	Univariate		Multivariate	
		*HR* (95%*CI*)	*P*	*HR* (95%*CI*)	*P*
Age (years)			0.518		–
>58	22	1		–	
≤58	18	1.242(0.643-2.398)		–	
Gender			0.952		–
male	35	1		–	
female	5	0.971(0.375-2.516)		–	
KPS			0.961		–
≥90	22	1		–	
<90	18	1.017(0.527-1.956)		–	
Interval time between BM and initial treatment (months)			0.309		–
≥8	12	1		–	
<8	28	1.485(0.690-3.196)		–	
Interval time between BM and end of chemoradiotherapy (months)			0.321		–
≥1.7	18	1		–	
<1.7	22	1.399(0.718-2.725)		–	
Brain metastases were found before PCI			0.883		–
No	26	1		–	
Yes	14	0.951(0.486-1.86)		–	
Multiple brain metastases			0.051		0.031
No	13	1		1	
Yes	27	2.012(0.983-4.118)		2.391(1.082-5.285)	
Symptomatic brain metastases			0.023		0.231
No	22	1		1	
Yes	18	2.118(1.091-4.109)		1.554(0.756-3.198)	
Extracranial disease control			0.710		–
Out of control	15	1		–	
Well controlled	25	0.881(0.450-1.721)		–	
Treatment of brain metastases					
No treatment	4	1		1	
Radiotherapy alone	23	0.151(0.047-0.483)	0.001	0.131(0.035-0.491)	0.003
Chemotherapy alone	4	0.339(0.081-1.408)	0.136	0.215(0.041-1.119)	0.068
Radiotherapy combined with chemotherapy	9	0.048(0.011-0.208)	<0.001	0.039(0.008-0.194)	<0.001

KPS, Karnofsky performance status; PCI, prophylactic cranial irradiation. -, uncompared.

## Discussion

SCLC patients are inclined to develop BM. At the initial diagnosis of SCLC, 10-20% of patients had BM, and 50-80% of patients would develop BM in the later course of disease (7). Studies have shown that PCI significantly reduced the incidence of BM and played a positive role in prolonging OS (8, 9). About 70-90% of patients will achieve a good response after definitive chemoradiotherapy, and PCI should be considered at this time (10). However, in clinical practice, some patients did not receive PCI for various reasons. Benjamin et al. reported that 44.7% of eligible LS-SCLC patients did not receive PCI, and the most common reason was the fear of neurotoxic side effects (38%). Other reasons included doctors’ opinion that it was inappropriate (33%) and advanced age (8%) (11). In our study, the main reason why patients did not receive PCI was also the fear of side effects. Nevertheless, currently, hippocampal avoidance during WBRT plus memantine has been shown to significantly reduce patients’ cognitive dysfunction (12).

In our study, 19 patients (30.2%) found BM before PCI by brain MRI. Chu et al. found that BM could be detected in 21.8% (24/110) of patients who achieved CR after definitive chemoradiotherapy before PCI, and the risk of BM increased with the extension of the interval time between the initial chemotherapy and MRI examination (13). The study of Manapov et al. showed that 32.5% (13/40) of LS-SCLC patients found BM by brain MRI before PCI (14). Thus, for LS-SCLC patients who achieved good response after definitive chemoradiotherapy, PCI should be administered as soon as possible to avoid BM (4). But there is no prospective study on the optimal timing of PCI.

For patients with BM without PCI, WBRT is still the current standard treatment, and the recommended dose is 30Gy in 10 fractions or 40Gy in 20 fractions. There is no significant impact of the two schedules on patients’ OS (15). Stereotactic radiosurgery is also an optional treatment for patients with limited BM (16). The study of Rusthoven et al. showed that the median OS of BM patients who have received WBRT was 5.2 months and that of the patients who have received stereotactic radiosurgery was 6.5 months after propensity matching analysis (17). In our study, the median OS of BM patients received radiotherapy alone was 10.6 months, which may be attributed to the better physical status of the patients included (KPS≥80). The study of Seute et al. implied that for SCLC patients receiving systemic chemotherapy, the response rate of extracranial metastases was 73%, while that of BM lesions was 27% (P=0.006). This might be the reason that the destruction of BM lesions to the blood-brain barrier was not enough to allow the penetration of chemotherapy drugs (18). This might be the reason why there was no statistically significant difference in OS between patients who received chemotherapy alone and those who did not receive anti-cancer treatment in our study. So, could combinations of different treatments improve the prognosis of BM patients? In a retrospective study included 698 SCLC patients with BM, patients received WBRT combined with chemotherapy (n=273) had a significantly better prognosis than those received supportive care alone (n=118) (P < 0.001) (19). However, several randomized controlled trials showed that there was no significant difference in OS between chemotherapy combined with WBRT and WBRT alone (20, 21). Another retrospective study included 101 SCLC patients with BM who received brain radiotherapy followed by chemotherapy, chemotherapy followed by brain radiotherapy, brain radiotherapy alone or chemotherapy alone, respectively. The results showed that there was a statistically significant difference in survival time between the chemotherapy followed by radiotherapy group and the chemotherapy alone group (22). In our study, radiotherapy combined with chemotherapy achieved better efficacy and significantly reduced the risk of death compared with monotherapy (radiotherapy combined with chemotherapy vs. radiotherapy alone, P=0.022; radiotherapy combined with chemotherapy vs. chemotherapy alone, P=0.025).

This study showed that multiple BM was an independent adverse prognostic factor for OS. The study of Varlotto et al. evaluated 137 BM patients, 38 of whom had multiple BM, and the median survival time of single and multiple BM patients were 23 months and 15 months respectively (P=0.004) (23). Hall et al. included 51 patients who survived more than 2 years in their study, single BM was significantly correlated with better OS (P < 0.0001) (24). In addition, over 50% of patients with extracranial disease progression may also affect OS (25). But in our study, the status of extracranial disease control had no significant impact on OS, possibly because of the limited sample size.

In conclusion, this study indicated that patients with BM secondary to SCLC had poor prognosis. Even if these patients received active radiotherapy/chemotherapy or even combined therapy, the OS remained significantly worse than other patients without BM.

The limitations of this study lie in its retrospective nature and the relatively small sample size, which may introduce bias. In addition, in this study, the frequency of brain MRI examination was not specified in the follow-up stage after treatment, but decided by the attending doctor according to the patient’s condition, which may result in the late detection of BM and adversely affect the prognosis of patients.

## Conclusion

LS-SCLC patients without PCI are very likely to develop BM and the prognosis would be dismal once BM was developed. Multiple BM is an adverse prognostic factor. Based on existing evidences, PCI is still a standard part of treatment for LS-SCLC patients with CR or PR after definitive chemoradiotherapy.

## Data availability statement

The raw data supporting the conclusions of this article will be made available by the authors, without undue reservation.

## Author contributions

QW and XH were responsible for the design of the study scheme, the implementation of the study and the writing of the paper; XH, FP, QZ, MYC, YK, YB and XY were responsible for the collection of clinical data and the proofreading of the paper; MC and XH guided and supervised the conduct of the experiment and the revision of the paper. All authors contributed to the article and approved the submitted version.

## References

[1] Govindan R Page N Morgensztern D Read W Tierney R Vlahiotis A. Changing epidemiology of small-cell lung cancer in the united states over the last 30 years: analysis of the surveillance, epidemiologic, and end results database. J Clin Oncol (2006) 24(28):4539–44. doi: 10.1200/JCO.2005.04.4859 17008692

[2] van Meerbeeck JP Fennell DA De Ruysscher DK. Small-cell lung cancer. Lancet (2011) 378(9804):1741–55. doi: 10.1016/S0140-6736(11)60165-7 21565397

[3] Turrisi AT 3rd Kim K Blum R Sause WT Livingston RB Komaki R. Twice-daily compared with once-daily thoracic radiotherapy in limited small-cell lung cancer treated concurrently with cisplatin and etoposide. N Engl J Med (1999) 340(4):265–71. doi: 10.1056/NEJM199901283400403 9920950

[4] Aupérin A Arriagada R Pignon JP Péchoux CL Gregor A Stephens RJ. Prophylactic cranial irradiation for patients with small-cell lung cancer in complete remission. prophylactic cranial irradiation overview collaborative group. N Engl J Med (1999) 341(7):476–84. doi: 10.1056/NEJM199908123410703 10441603

[5] National Comprehensive Cancer Network (NCCN). NCCN clinical practice guidelines in oncology: Small cell lung cancer. v. 3 (2023). Available at: https://www.nccn.org/professionals/physician_gls/pdf/nscl.pdf.

[6] Wolfson AH Bae K Komaki R Meyers C Movsas B Pechoux CL. Primary analysis of a phase II randomized trial radiation therapy oncology group (RTOG) 0212: Impact of different total doses and schedules of prophylactic cranial irradiation on chronic neurotoxicity and quality of life for patients with limited disease small-cell lung cancer. Int J Radiat Oncol Biol Phys (2011) 81:77–84. doi: 10.1016/j.ijrobp.2010.05.013 20800380 PMC3024447

[7] Quan AL Videtic GM Suh JH. Brain metastases in small cell lung cancer. Oncology (2004) 18(8):961–72. 15328892

[8] Meert AP Paesmans M Berghmans T Martin B Mascaux C Vallot F. Prophylactic cranial irradiation in small cell lung cancer: a systematic review of the literature with meta-analysis. BMC Cancer (2001) 1:5. doi: 10.1186/1471-2407-1-5 11432756 PMC34096

[9] Kalemkerian GP. Small cell lung cancer. Semin Respir Crit Care Med (2016) 37(5):783–96. doi: 10.1055/s-0036-1592116 27732999

[10] Wang A Zimmermann S Parikh K Mansfield AS Adjei AA. Current diagnosis and management of small-cell lung cancer. Mayo Clin Proc (2019) 94(8):1599–622. doi: 10.1016/j.mayocp.2019.01.034 31378235

[11] Lok BH Ma J Foster A Perez CA Shi W Zhang Z. Factors influencing the utilization of prophylactic cranial irradiation in patients with limited-stage small cell lung cancer. Adv Radiat Oncol (2017) 2(4):548–54. doi: 10.1016/j.adro.2017.08.001 PMC5707415 29204521

[12] Brown PD Gondi V Pugh S Tome WA Wefel JS Armstrong TS. Hippocampal avoidance during whole-brain radiotherapy plus memantine for patients with brain metastases: Phase III trial NRG oncology CC001. J Clin Oncol (2020) 38(10):1019–29. doi: 10.1200/JCO.19.02767 PMC7106984 32058845

[13] Chu X Li S Xia B Chu L Yang X Ni J. Patterns of brain metastasis immediately before prophylactic cranial irradiation (PCI): implications for PCI optimization in limited-stage small cell lung cancer. Radiat Oncol (2019) 14(1):171. doi: 10.1186/s13014-019-1371-4 31533763 PMC6749639

[14] Manapov F Klautke G Fietkau R. Prevalence of brain metastases immediately before prophylactic cranial irradiation in limited disease small cell lung cancer patients with complete remission to chemoradiotherapy: a single institution experience. J Thorac Oncol (2008) 3(6):652–5. doi: 10.1097/JTO.0b013e3181757a76 18520807

[15] Früh M Ruysscher DD Popat S Crinò L Peters S Felip E. Small-cell lung cancer (SCLC): ESMO clinical practice guidelines for diagnosis, treatment and follow-up. Ann Oncol (2013) 24(Suppl 6):vi99–105. doi: 10.1093/annonc/mdt178 23813929

[16] Chi A Komaki R. Treatment of brain metastasis from lung cancer. Cancers (2010) 2(4):2100–37. doi: 10.3390/cancers2042100 PMC3840463 24281220

[17] Rusthoven CG Yamamoto M Bernhardt D Smith DE Gao D Serizawa T. Evaluation of first-line radiosurgery vs whole-brain radiotherapy for small cell lung cancer brain metastases: The FIRE-SCLC cohort study. JAMA Oncol (2020) 6(7):1028–37. doi: 10.1001/jamaoncol.2020.1271 PMC7273318 32496550

[18] Seute T Leffers P Wilmink JT Velde GPMT Twijnstra A. Response of asymptomatic brain metastases from small-cell lung cancer to systemic first-line chemotherapy. J Clin Oncol (2006) 24(13):2079–83. doi: 10.1200/JCO.2005.03.2946 16648509

[19] Li H Xue R Yang X Han S Yang W Song X. Best supportive care versus whole-brain irradiation, chemotherapy alone, or WBRT plus chemotherapy in patients with brain metastases from small-cell lung cancer: A case-controlled analysis. Front Oncol (2021) 11:568568. doi: 10.3389/fonc.2021.568568 33732638 PMC7957068

[20] Neuhaus T Ko Y Muller RP Grabenbauer GG Hedde JP Schueller H. A phase III trial of topotecan and whole brain radiation therapy for patients with CNS-metastases due to lung cancer. Br J Cancer (2009) 100(2):291–7. doi: 10.1038/sj.bjc.6604835 PMC2634726 19127261

[21] Postmus PE Haaxma-Reiche H Smit EF Groen HJ Karnicka H Lewinski T. Treatment of brain metastases of small-cell lung cancer: comparing teniposide and teniposide with whole-brain radiotherapy–a phase III study of the European organization for the research and treatment of cancer lung cancer cooperative group. J Clin Oncol (2000) 18(19):3400–8. doi: 10.1200/JCO.2000.18.19.3400 11013281

[22] Liu Y Liu X Wang Y Zhu J Xin Y Niu K. A study on different therapies and prognosis-related factors for 101 patients with SCLC and brain metastases. Cancer Biol Ther (2017) 18(9):670–5. doi: 10.1080/15384047.2017.1360450 PMC5663412 28812423

[23] Varlotto JM Flickinger JC Niranjan A Bhatnagar AK Kondziolka D Lunsford LD. Analysis of tumor control and toxicity in patients who have survived at least one year after radiosurgery for brain metastases. Int J Radiat Oncol Biol Phys (2003) 57(2):452–64. doi: 10.1016/S0360-3016(03)00568-6 12957257

[24] Hall WA Djalilian HR Nussbaum ES Cho KH. Long-term survival with metastatic cancer to the brain. Med Oncol (2000) 17(4):279–86. doi: 10.1007/BF02782192 11114706

[25] Vuong DA Rades D Vo SQ Busse R. Extracranial metastatic patterns on occurrence of brain metastases. J Neurooncol (2011) 105(1):83–90. doi: 10.1007/s11060-011-0563-z 21394486

